# Research progress of neuroblastoma related gene variations

**DOI:** 10.18632/oncotarget.14408

**Published:** 2016-12-31

**Authors:** Yanna Cao, Yan Jin, Jinpu Yu, Jingfu Wang, Jie Yan, Qiang Zhao

**Affiliations:** ^1^ Department of Pediatric Oncology, Tianjin Medical University Cancer Institute and Hospital, National Clinical Research Center for Cancer, Key Laboratory of Cancer Prevention and Therapy of Tianjin, Tianjins Clinical Research Center for Cancer, Tianjin, P.R. China; ^2^ Department of Cancer Molecular Diagnostic Center, Tianjin Medical University Cancer Institute and Hospital, National Clinical Research Center for Cancer, Key Laboratory of Cancer Prevention and Therapy of Tianjin, Tianjins Clinical Research Center for Cancer, Tianjin, P.R. China

**Keywords:** neuroblastoma, gene, high throughput nucleotide sequencing

## Abstract

Neuroblastoma, the most common extracranial solid tumor among children, is an embryonal tumor originating from undifferentiated neural crest cell. Neuroblastomas are highly heterogeneous, represented by the wide range of clinical presentations and likelihood of cure, ranging from spontaneous regression to relentless progression despite rigorous multimodal treatments. Approximately, 50% of cases are high-risk with overall survival rates less than 40%. With the efforts to collect large numbers of clinically annotated specimens and the advancements in technologies, researchers have revealed numerous genetic alterations that may drive tumor growth. However, the most lack mutations in genes that are recurrently mutated, which inspires researchers to identify disrupted pathways instead of single mutated genes to unearth biological systems perturbed in neuroblastoma. Stratification of patients and target therapy based on their molecular signatures have been the center of focus. This review provides a comprehensive summary of the recent advances in identification of candidate genes variations, targeted approaches to high-risk neuroblastoma and evaluates the methods utilized for detection, which will provide new avenues to develop therapies and further genetic researches.

## INTRODUCTION

Neuroblastoma(NB), a childhood cancer of the developing sympathetic nervous system, is the most common pediatric solid tumor, accounting for approximately 7% of childhood malignancies and 15% of pediatric oncology deaths [1]. According to the Cancer Statistics Review of Surveillance, Epidemiology, and End Results Program (SEER) conducted by the United States, more than 650 cases are diagnosed each year in North America [2, 3]. The incidence is about 10.54 cases per 1 million per year in children younger than 15 years [4, 5]. Prognosis of NB is associated with a number of factors, including International Neuroblastoma Risk Group (INRG) staging, age at diagnosis, histopathological classification, degree of tumor differentiation, amplification of N-MYC, loss of heterozygosity of 11q and DNA ploidy [6]. Based on the above factors, neuroblastoma patients can be classified into 4 groups: extremely lowrisk , lowrisk , moderaterisk , and highrisk [6]. Patients with low or intermediate risk can achieve an overall survival (OS) rate greater than 95% with surgery alone [7]. Furthermore, some studies have shown that infants with localized tumors can be cured without any treatment, including surgery [8, 9]. Approximately 50% of cases are high-risk with overall survival rates less than 40% [10]. High-risk patients often go through rigorous treatment consisting of 3 treatment blocks: Induction (chemotherapy and tumor resection), consolidation (high-dose chemotherapy with autologous stem-cell rescue and external-beam radiotherapy) and post-consolidation (anti-ganglioside 2 immunotherapy with cytokines and cis-retinoic acid) [7]. Heterogeneity is a clinical hallmark of NB, represented by its wide range of clinical behaviors and diverse response to treatments [11]. A subset of tumor will undergo spontaneous regression; while others will progress relentlessly into high-risk metastatic disease with poor prognosis despite the use of multimodal intensive treatment. Such diversity can be attributed to molecular differences [11]. Currently, molecular signature only constitutes a small portion of parameters used for prognostic evaluation, including MYCN amplification, 11q absence and DNA ploidy. However other molecular oriented parameters, such as mutations in ATRX, ALK, and variations of chromosome fragments, such as 1p and 17q, have not yet been included in the evaluation systems of NB. The most likely reason is that neuroblastomas show some mutations (mentioned below), but the most lack mutations in genes that are frequently mutated [12]. Surprisingly, some studies have shown high-risk NB was associated with fewer recurrent somatic mutations [13]. And compared with adult cancers, neuroblastomas show lower number of activating mutations affect protein functions [12].

This review primarily focuses on both germline mutations predisposing children to the development of neuroblastoma and somatic events associated with neuroblastoma pathogenesis and clinical phenotypes. Through the comprehensive summary, it may provide some tips in the studies of genetic studies or targeted therapies for neuroblastoma.

## GERMLINE MUTATIONS OF NB

Recent studies have shown that germline mutations of NB can be classified into two types, namely familial, and sporadic genetic susceptible genes.

### Familial NB

Familial NB, primarily featuring rare mutations of certain genes, merely accounts for 1 percent of all the NB patients [14]. Familial NB is incomplete explicit autosomal dominant inheritance. In contrast to sporadic cases, familial cases occur at a younger age, related to multifocal primary tumors [15]. According to the literatures, germline mutations in familial NB mainly occur in two genes, namely paired-like homeobox 2B (PHOX2B) and anaplastic lymphoma kinase (ALK).

PHOX2B, the first identified predisposition gene related to NB [16], locates in 4p12 area of human chromosome, which encodes and regulates transcription factor of neural crest development. Most of patients harboring PHOX2B mutations are accompanied by complications, including congenital centrum hypopnea syndrome, congenital megacolon, multiple neurofibromatosis and pheochromocytoma [17, 18]. Several mutations in the PHOX2B have been identified in sporadic and familial neuroblastoma [17]. These mutations are believed to interfere with the PHOX2B protein's role in promoting nerve cell differentiation. However, germline mutations of PHOX2B accounted for merely 6.4 % of hereditary neuroblastoma cases and were rarely detected in more common sporadic cases of the disease, indicating that the gene was not the major pathogenic gene [17, 19].

ALK gene mutations are more common than PHOX2B gene mutations in familial NB. ALK gene lies in human chromosome 2p23, encoding tyrosine kinase receptor that belongs to insulin receptor superfamily. It plays an important role in brain and specific neuron development. Amplification or mutation of ALK promotes phosphorylation of ALK, leading to increased kinase activity and ultimately results in tumorigenesis [20, 21]. In familial NB, ALK mutations often occur in coding areas, such as hotspots F1174, F1245 and R1275. Among these loci, the R1275Q mutation is the most common germline ALK mutation, occurring in about 45% of hereditary neuroblastomas [22]. These mutations also occur in the somatic cells, the most common somatic mutation in neuroblastoma, accounting for 6%-12% of sporadic neuroblastoma [12, 20, 23–28]. Among these mutations, ALK R1275Q is also the most common somatic ALK alteration [29, 30]. In addition, F1174L mutants are observed in a higher frequency in MYCN-amplified tumors [31], suggesting their pathogenic association [32]. ALK interacts with MYCN *via* multiple mechanisms: ALK was found to induce the expression of MYCN by promotor activation [33] and activation through ERK5 [34]; In addition, phosphoinositide 3-kinase/AKT further activates the ALK downstream signaling controlling glycogen synthase kinase 3 beta activity and MYCN protein stabilization. The observed synergistic effect of that mutant ALK accelerated tumor formation in MYCN transgenic mice [32, 35, 36] could be explained by the above mechanism.

With the knowledge of familial NB, recent years some researchers have proposed that screening of germline mutation of ALK and PHOX2B for sick children with NB family history or two-sided adrenal gland goiter should be performed. If ALK or PHOX2B gene was mutated, the sick children should undergo abdomen ultrasound and urine catecholamine levels detection every three months until the sick children reach 5 years old even though they are asymptomatic [37]. However, A recent study from pediatric cancer genome project suggested that less than 8.5% of pediatric cancer patients including neuroblastoma could be detected germline mutated predisposition genes. In addition, only 40% of their patients with germline mutations were pathogenic or probably pathogenic and that could be evaluated had a family history of cancer [38]. Derived from these, family history did not predict the presence of an underlying predisposition syndrome in most patients. This conclusion challenges the conventional belief for family history of NB.Thus, due to lack of enough evidences, screening of sick children with familial NB has yet to be further improved.

### Sporadic NB

Sporadic neuroblastoma is more common. Through using genome-wide association studies (GWAS), several common genomic variables (single nucleotide polymorphisms [SNPs] and copy number variations [CNVs]) associated with sporadic neuroblastoma have been identfied.These genomic variables could be classified into three categories, among which two categories were related to NB risks. The first category was found in NB patients with high risk, including CASC15/14, BARD1, LMO1, LIN28B and HACE1 [37, 39]. The second category occurred in NB patients with low risk, including genes DUSP12 、 DDX 、 IL31RA and HSD17B12 [40]. The third category was variation of germline copy numbers, for example, gene NBPF23 [41].

Maris et al. identified common SNPs at 6p22 within CASC15 and CASC14 genes associated with neuroblastoma risk. Homozygosity for the at-risk G allele of rs6939340, the most significantly associated SNP, has an increased risk of developing neuroblastoma with odds ratio of 1.97. These gene variations were more likely to occur among stage 4 NB patients and individuals who carried MYCN amplification [42].

Several SNPs in BRCA1 associated RING domain 1 (BARD1) located in the area 2q35 of human chromosome were related with invasive NB. The function of the protein encoded by gene BARD1 is to form heterodimer in conjunction with BRCA1 protein. Stable formation of the heterodimer may be critical for BRCA1 exerting cancer inhibition [43]. A study comparing 397 high-risk cases and 2,043 controls revealed six new SNPs at 2q35 within the BARD1 gene locus significantly associated with NB [43]. They show that common variation in BARD1 associates with the risk of the aggressive and most clinically corresponding subtype of human neuroblastoma. Furthermore, Pugh et al. tested tumor tissue DNA and the matching peripheral blood DNA by using the next sequence, discovering gene BARD1 could undergo germline mutations (c.334C > T, c.1921C > T) [12].

The LMO1 risk alleles and copy-number gains are associated with increased LMO1 expression in NB primary tumors and cell lines, consistent with a gain of functional role in tumorigenesis [44]. The protein coded by gene LMO1 was transcription factor, including two LIM areas rich in cysteine, which played a role in protein interactions. Previous studies also indicate that LMO1 participates in regulation of nerve system development [45]. In the first GWAS performed on Chinese children, Wang et al. found that LMO1 on chromosome 11p15.4 was related to susceptibility of NB. They conducted NB GWAS including 549 cases (244 NB patients and 305 healthy controls). Eleven SNPs located within LMO1 were found significantly associated with NB, and rs204926 was confirmed to have the closest relationship [39]. At the somatic level, the LMO1 is mutated in 12% of NB, primarily through duplication, and this is associated with disease progression and poor survival [44].

#### LIN28B

Given the central role of MYCN in neuroblastoma biology, understanding its upstream regulators is also important. LIN28B has been shown to positively regulate MYCN levels through let-7 binding [2]. MYCN indirectly affects the expression of LIN28B through regulating miR-26a-5p( MiR-26a-5p and miR-26b-5p regulate LIN28B expression), and directly regulates LIN28B expression through a binding site within LIN28B promoter [46]. Taken together, these data points to a complex reciprocal regulatory relationship between the two genes [47]. LIN28B-let-7-MYCN regulation and control system blocked the differentiation of normal neuroblasts cells [48].

Besides common SNP variations, neuroblastoma was the first tumor that germline copy number variation was found to contribute to its susceptibility [41]. NBPF23 (Neuroblastoma breakpoint family, member 23) is located on chromosome 1q21.1 harboring a region where varies in copy number among population. Furthermore hemizygous deletion of this region is significantly associated with neuroblastoma [37]. The neuroblastoma breakpoint family (NBPF) has been found to play regulatory roles in neuroblastoma development and human evolution. However, the mechanism for the regulation and function of this family is still unknown. NBPF may function as DNA-binding transcription factor in nucleus, which provides important new insights into the functions of NBPF genes in the human cells [49].

### Other germline mutations related to NB

DNA repair genes. DNA repair genes have been reported in oncogenesis of multiple cancers including neuroblastoma, such as BRCA1/2, PALB2, FANCD2 and CHEK2 et al. [50–55]. Pugh et al. performed whole genome sequencing of peripheral blood DNA samples on 240 cases of NB patients, and found a few germline mutations including CHEK2 (c.433C > T, c.542G > A and c.539G > A), PINK1 (c.1040T > C and c.836G > A), BARD1(c.334C > T and c.1921C > T) and PALB2 (c.1684+1C > A) [12]. Brooks et al. reported 20 cases of pediatric cancer including neuroblastoma among 379 families, uncovering mutations in either BRCA1 or BRCA2 [54]. Other studies also identified a deletion/insertion in the FANCD2 gene in nephro- and neuroblastomas [55]. Particularly, germline mutations in BRCA2 and PALB2, that PALB2 binds to the N terminus of BRCA2 and has a key role in localization and stabilization of BRCA2, have been detected that they were associated with the development of neuroblastoma [51, 52].

#### STK11

Papillary thyroid carcinoma (PTC), known as a secondary malignancy after treatment for neuroblastoma (NB), is rare in children. Targeted next-generation sequencing analysis for a 46 cancer-gene profile was performed on both tumors and peripheral blood DNA. A heterozygous missense mutation in STK11 (F354L) was identified in both NB and PTC. This mutation was also detected in peripheral blood cells [56]. However, the role of this gene in NBs requires further study.

## SOMATIC MUTATIONS IN NB

Somatic events of NB can be classified as copy number variations and somatic gene mutations.

### Copy number variations

Copy number variant (CNV) is an important and major source of variation in the human genome, comprising of large insertions and deletions that lead to gain or lose segments of chromosomes. Traditional genome-wide approaches to detect CNVs make use of single nucleotide polymorphism (SNP) array data or array comparative genome hybridization (aCGH) [57–59]. The minimum detectable size and breakpoint resolution are limited, for all results relying on the density of probes on the array. NGS offers higher sensitivity, and is a cost-effective alternative for CNV testing [60–62]. Copy number variation is relative to the structure variation of genome, including MYCN and other genes amplifications and chromosomal gains and losses. Comparing to adult tumors, the number of genetic somatic mutations in neuroblastoma was low, but the frequency of recurrent copy number variations was relatively high. Therefore CNVs could be used as biomarkers for neuroblastoma [26].

### Somatic DNA amplification

DNA amplification plays a critical role in the development of solid tumors, potentially by causing overexpression of oncogenes. MYCN was the first proto-oncogene found to be amplified with significant clinical relevance, and its status was routinely used to direct treatment. As mentioned above, genes like LIN28B and ALK have been shown to regulate MYCN levels through certain mechanisms, bringing the old enemy into the focus of current and future targeted drug efforts.

MYCN, amplification of c-MYC homolog, has been shown to strongly correlate with poor prognosis [32, 63], considered as the best-characterized biomarker of risk for NB [63, 64]. It is located at chromosome 2p24 [65] and plays an important role in shortening cell cycle, promoting cell proliferation, inhibiting cell differentiation and apoptosis. Amplification of MYCN (no less than 10 times duplication of diploid genome or greater than 4 times duplication of number 2 chromosome related to signal) accounts for about 22% of gross occurrence of NB, mostly co-existing with other perilous factors [66].

Other regional amplifications. Previous studies have also reported other amplifications, such as ALK, DDX1 and OCD1 amplifications, associated with low recurrence and often co-occurring with MYNC amplification. Apart from MYCN, ALK is the most frequently amplified gene, accounting for 4% of NBs [23–25], followed by DDX1, located in close vicinity of MYCN [67]. ODC1 amplification at band 2p25, somatic amplification at 12q13-15 locus containing CDK4 (12q13_14) and MDM2 (12q15) were always found to be co-amplified with MYCN [26, 68, 69]. The NAG gene mapped in close vicinity to MYCN on chromosome band 2p24 was also found to be co-amplified with MYCN [70]. However, in the cases with absence of MYCN amplification, the amplicons at 12q13-14 and 12q13-15 , and CDK6 gene at 7q21, CCND1 gene at 11q13 were also detected [69]. Surprisingly, most of these genes are involved in cell cycle. This suggests that cell cycle regulators could play a role in neuroblastoma tumorigenesis.

### Somatically chromosomal gains and losses

Somatically chromosomal imbalance is a key feature of NB: they occur as genomic amplifications or deletions as well as whole or segmental chromosome imbalances. Many chromosome alterations (whole chromosome gains or losses), resulting in hyperdiploidy, are associated with low-risk disease and favorable outcomes, while segmental chromosomal alterations (deletions of chromosome arms 1p, 3p, 4p, 9p, 11q and gains of chromosome arms 1q, 2p, 17q) are associated with more aggressive diseases [16, 71].

### Loss of 1p and 11q

Loss of heterozygosity at chromosome 1p36, occurring in 23%-35% patients, is associated with other high-risk clinical and genomic features, such as older age, MYCN amplification, and metastatic disease [72–75]. Compared to the tumors without MYCN amplification, tumors with MYCN amplification generally had larger 1p deletions (the median size of deletion for MYCN-amplified tumors was 84 Mb and for non-amplified 46 Mb) [76]. Genes located at chromosome 1p36 including CHD5, CAMTA1 and PIK3CD are mostly likely to be deleted in the tumor [77–79]. CHD5 was first identified as a candidate tumor suppressor gene(TSG) which was frequently deleted in 1p36.31 of NBs [80]. High CHD5 is strongly related to favorable clinical, biological features and outcome. On the contrary, low/absent expression is associated with older age, higher stage, MYCN amplification and a poor outcome [81, 82]. Loss of chromosome 11q, most commonly seen in tumors without MYCN amplification, occurs in approximately 33% of neuroblastomas and is associated with a poor prognosis [72]. The related genes located at chromosome 11q include CADM1 and ATM [83, 84]. CAMTA1 qualifies as a TSG in NB. Low CAMTA1 expression is associated with unfavorable features (advanced stage, MYCN amplification) and poor outcome [79, 85].

### 17q gain

The most frequently identified genomic alteration of neuroblastoma cells is somatic gain of the distal portion of chromosome 17q, which occurs in at least half of primary tumors, predicting an overall poor prognosis, and frequently associated with other parameters of aggressive disease such as older age, MYCN amplification, and chromosome 1p deletion [86, 87]. At least two genes, survivin/BIRC5 and nm23/NME1, mapping to 17q gain regions, have been implicated contributing to the aggressive phenotype of neuroblastomas. Survivin/BIRC5 is an anti-apoptotic protein, and its expression is associated with poor prognosis, and nm23/NME1 encodes a nucleoside diphosphate kinases, involved in cell proliferation and differentiation. Overexpression of NME1 is associated with unfavorable outcome and aggressive features [79, 88, 89].

### Loss of 9p and 3p

Caren et al. employed SNPs chip to analyze NB samples, and found chromosome 9p might undergo homozygous or heterozygosis loss. The genes involved included CDKN2A and CDKN2B. A region of homozygous deletion was discovered in one NB tumor sample, located in chromosome 3p24.1, harboring the gene RBMS3. They also detected two homozygous deletions in a NB cell line Kelly, one in chromosome 3p, covering the gene LSAMP, the other in gene PTPRD in chromosome 9p [76].

### Somatic mutations of NB

Compared with adult tumors, mutation frequencies of tumor cell of children are relatively lower [90], but activating mutations frequently affect specific biological processes in aggressive neuroblastoma [91].

ATRX, a gene plays an important role in epigenetic regulation, was found to be mutated in approximately 50% of adolescent and young adults with neuroblastoma [28, 92]. Although located in chromosome X, ATRX mutations were found in both males and females, consistent with previous reports [28]. Putative genetic loss-of-function alterations in the ATRX gene have been identified in nearly 10% of neuroblastomas [12, 27], and enriched in older patients [28]. In infant tumors or in tumors with MYCN amplification, no ATRX mutations are found, indicating ATRX alterations occur in a subtype of NB. Although the mechanism is poorly understood, neuroblastoma tumors with ATRX loss of function mutations were found to have lengthened telomeres. Nevertheless, these findings suggest that anti-telomerase-based therapies might benefit neuroblastoma patients with ATRX mutations [21, 93].

Other genes involved in chromatin regulation. Protein products encoded by gene ARID1A located at chromosome 1p35.3 and gene ARID1B located at chromosome 6q25.1 are SWI/SNF family members [94]. They regulate gene expression by modifying chromatin structure. Mark Sausen et al. [26] detected gene ARID1A could undergo nonsense, missense, disconnection mutation, and heterozygosity deficiency of somatic cell; ARID1B could suffer heterozygosity deficiency and point mutation of somatic cell. They contributed to failures of early stage treatment for NB patients and low survival rate to mutations of the two genes, suggesting ARID1A and ARID1B as contributors to neuroblastoma oncogenesis [26]. Interestingly, several sequence alterations in other genes involved in chromatin regulation in neuroblastoma have been found, including EP300, CREBBP, TTF2, KDM5A, CHD9, and gene IKZF1 [91], which might undergo somatic mutations and promote NB occurrence [44]. Thus, Chromatin remodeling may play an important role in the occurrence of NB.

PTPN11, locates at chromosome 12q24, encoding protein SHP-2, has an important role in signal transduction downstream of growth factor receptor signalling. Activating mutations of PTPN11 have been associated with developmental pathologies in neuroblastoma. Through activation of the RAS-ERK signalling pathway, SHP2 is ubiquitously expressed and regulates cell survival and proliferation. Therefore, reduction of SHP2 activity to suppress tumour cell growth is a potential target of cancer therapy [95–97].

NTRK1 encodes TrkA, which is a high affinity nerve growth factor (NGF) receptor. TrkA is involved in neural crest cell differentiation, and its expression has been reported to be associated with a favourable prognosis in neuroblastoma [98, 99]. NTRK1 has been detected in NB to undertake c.1810C > T mutation, which is common in NB for baby younger than 18 months without MYCN multiplication [100].

## PROMISING THERAPEUTIC TARGETS

With the efforts to collect large numbers of clinically annotated specimens and the advancements in technologies, researchers have discovered numerous therapeutic targets and subsequently developed agents to target them. Although the most frequent alteration in neuroblastoma is the amplification of MYCN, it's difficult to target MYCN directly. As an alternative to target MYCN, we could target effector molecules downstream/upstream of MYCN in MYCN-related signaling pathways, such as the p53/MDM2/p14 (ARF) pathway, WNT/β-catenin pathway, and PI3K/mTOR pathway [64, 79, 101–107]. Some studies have shown that PI3 or Aurora A kinase inhibition can destabilize MYCN protein. Currently, an Aurora A kinase inhibitor is being evaluated in combination with irinotecan and temozolomide in a phase I clinical trial [7]. ALK alterations may arise in relapsed patients [108], occupying the position after the MYCN in the NB candidate genes. Crizotinib is a dual ALK/MET inhibitor, approved by the US food and drug administration (FDA) for patients with NSCLC harboring ALK rearrangements. However, a phase I clinical trial evaluating the efficacy of crizotinib as a single-agent revealed unfavorable results in neuroblastoma patients [109]. Therefore, the proportion of ALK-positive neuroblastoma patients benefits from Crizotinib is limited [110–112]. ATRX was found to be mutated in approximately 50% of adolescent and young adults with neuroblastoma [28, 92]. Preclinical and clinical investigations are required to prove the efficacy of ATRX inhibitors in this group of patients. The identification of carcinogenic mutations and an increased understanding of how these mutations trigger tumorigenesis enable researchers to develop agents to target such alteration.

## CONCLUSIONS AND PERSPECTIVES

In summary, the research on the pathogenesis of NB is facing a great challenge for its extremaly low mutation frequency. Occurrence of NB may be a multi-stage process with the simultaneous activation of multiple carcinogenic signalling routes, suggesting that growing interest should be stimulated in identifying disrupted pathways, instead of single mutated genes, thereby unearthing biological systems perturbed in neuroblastoma. There are a number of proteins and pathways that are relevant to NB pathogenesis. Major genes and pathways include (Table 1): a) The signaling pathway network common to many RTKs and other intracellular kinases, represented by RAS, RAF, PI3K, MAPK, MEK, AKT and mTOR [113]. These signal routes lead to cancer *via* mechanisms such as apoptosis, multiplication, DNA repair, cancer cell transfer and angiogenesis. The RAS pathway is among the most frequently mutated pathways in human cancer. Studies showed that RAS mutations could be predictive markers for treatment in neuroblastomas [114]; b) Embryonic development-related signaling pathways, such as Wnt, Notch and Hedgehog [115]. Taking NOTCH signal as an example, inhibiting Notch signal leads to NB cells differentiate more maturely [116]. At the early stage of nerve development, Notch signalling route may regulate multiplication, differentiation and apoptosis of cells, which may take part in generation of tumor angiogenesis through connecting to signal PI3K-AKT [117]. The Notch pathway may be activated by the homeobox transcription factor PHOX2B [118].

**Table 1:** Classification of functions of candidate genes relating to NB

Pathways and functions	Gene list
*The signaling pathway network common to many RTKs and other intracellular kinases*	*BRAF, PTPN11, PDGFRA, HRAS, KRAS, NRAS, NF1, KIT, TIAM1, MDM2, PIK3CA, ERBB2, FGFR4, TP53, EGFR, PTEN, NTRK1, CDKN2A/B, CDKN1C, PTPRD, MYCN, ALK, CDK4/6, CHEK2, RB1, RBMS3, CCND1, BARD1,BCOR, PINK, BRCA2, FANCA, PALB2*
*Multiplication and apoptosis*	*PTPN11, ALK, CDK4/6, CHEK2, RB1, PTEN CDKN2A/B PTPRD, CDKN1C, MYCN, RBMS3*
*DNA repair*	*BRCA2, FANCA, CHEK2, PALB2*
*cancer cell transfer and angiogenesis*	*TIAM1, RBMS3*
*Embryonic development related signaling pathway*	*APC AXIN2 CTNNB1, FBXW7, PHOX2B, OS9 GLI1 PTCH1, LMO1*
*Signaling by WNT*	*APC AXIN2 CTNNB1*
*Signaling by NOTCH*	*PHOX2B, FBXW7, TP53, CCND1*
*Signaling by Hedgehog*	*PTCH1, OS9, GLI1*
*The possible genes related to MYCN*	*ALK, LIN28, ODC1, CDK4, MDM2 , DDX1, NAG and other molecules downstream/upstream of MYCN in MYCN-related signaling pathways*
*Chromatin remodeling*	*ARID1A/B, ATRX*

Since 2005, GWAS has been frequently used for tumor studies by means of comparison between different frequencies of SNP in cases and controls to seek risk mutations. However , GWAS is not a powerful approach for studying rare or uncommon SNPs, because GWAS results tend to have problems such as false positive, false negative, only a few single nucleotide polymorphisms detected locate in functional areas, and insensibility to rare mutation and structure mutation, leading to limitation of its application [119]. The paradigm of DNA next generation sequencing(NGS) could process millions of DNA templates in parallel, resulting in a low cost per base and a throughput on the gigabase (Gb) scale [120]. Progress of sequencing technique of new generation promotes fast development of whole genome sequencing, whole genome exon sequencing and depth sequencing of target genes, which is possible to solve the above-mentioned problems. Moreover, according to the characteristics of gene variants in neuroblastoma, perhaps the main power of NGS was the possibility to combine both mutation and copy-number events, to generate broad cataloging of genetic variations in neuroblastomas. However, the biggest challenge for us is that we must be able to distinguish functionally relevant mutations from nonpathogenic variants. This distinction is critical not only to correlate these with oncogenic potency, but also to deliver to the research of pathogenesis of NB. Thus, deep resequencing of target genes captured is a key method to locate potential pathopoiesia mutation of neuroblastoma, featuring high degree of specificity and feasibility, which provides potential new target spots for therapy intervening measure.
